# A review on the role of CASC11 in cancers

**DOI:** 10.3389/fcell.2023.1131199

**Published:** 2023-06-23

**Authors:** Soudeh Ghafouri-Fard, Atefeh Harsij, Bashdar Mahmud Hussen, Mohammad Taheri, Guive Sharifi

**Affiliations:** ^1^ Department of Medical Genetics, School of Medicine, Shahid Beheshti University of Medical Sciences, Tehran, Iran; ^2^ Phytochemistry Research Center, Shahid Beheshti University of Medical Sciences, Tehran, Iran; ^3^ Department of Clinical Analysis, College of Pharmacy, Hawler Medical University, Erbil, Iraq; ^4^ Institute of Human Genetics, Jena University Hospital, Jena, Germany; ^5^ Urology and Nephrology Research Center, Shahid Beheshti University of Medical Sciences, Tehran, Iran; ^6^ Skull Base Research Center, Loghman Hakim Hospital, Shahid Beheshti University of Medical Sciences, Tehran, Iran

**Keywords:** CASC11, lncRNA, cancer, expression, biomarker

## Abstract

The long non-coding RNA (lncRNA) cancer susceptibility 11 (CASC11) is a newly identified lncRNA located on chromosome 8q24.21. The expression of lncRNA CASC11 has been found to be elevated in different cancer types and the prognosis of the tumor is inversely correlated with the high CASC11 expression. Moreover, lncRNA CASC11 has an oncogenic function in cancers. The biological characteristics of the tumors, such as proliferation, migration, invasion, autophagy, and apoptosis can be controlled by this lncRNA. In addition to interacting with miRNAs, proteins, transcription factors, and other molecules, the lncRNA CASC11 modulates signaling pathways including Wnt/β-catenin and epithelial-mesenchymal transition. In this review, we have summarized studies on the role of lncRNA CASC11 in the carcinogenesis from cell lines, *in vivo*, and clinical perspectives.

## Introduction

According to the ENCODE project, although more than 80% of the human genome is transcribed, about 98% of these transcripts do not encode proteins (Harrow et al., 2012). A particular type of RNAs, called long non-coding RNAs (lncRNAs) lacks the ability to code for proteins but are involved in important cellular processes (Bridges et al., 2021). LncRNAs appear to play a variety of roles in the regulation of epigenetic modifications, transcription, post-transcriptional modifications, and translation, according to numerous studies that have been conducted up to now (Bhat et al., 2016; Peng et al., 2017). They can interact with proteins while still being linked to their transcriptional site or they can interact with chromatin-modifying complexes to regulate transcription of target genes in *cis* or *trans*, respectively (Rinn et al., 2007; Wang et al., 2008). In addition, the possibility of lncRNAs interacting with microRNAs (miRNAs) to carry out their biological functions has long been known (Jalali et al., 2013). Undeniably, lncRNAs are involved in the pathogenesis of many diseases, including various cancers (Chen F. et al., 2019).

Different functions of lncRNAs depend on their localization and their specific interfaces with DNA, RNA and proteins. Through these interactions, lncRNAs regulate chromatin function and modulate the establishment and function of membraneless nuclear bodies. Most notably, lncRNAs can change the stability and translation of mRNAs in the cytoplasm. Similar to protein coding genes, lncRNAs interfere with signaling pathways (Statello et al., 2021).

**FIGURE 1:**
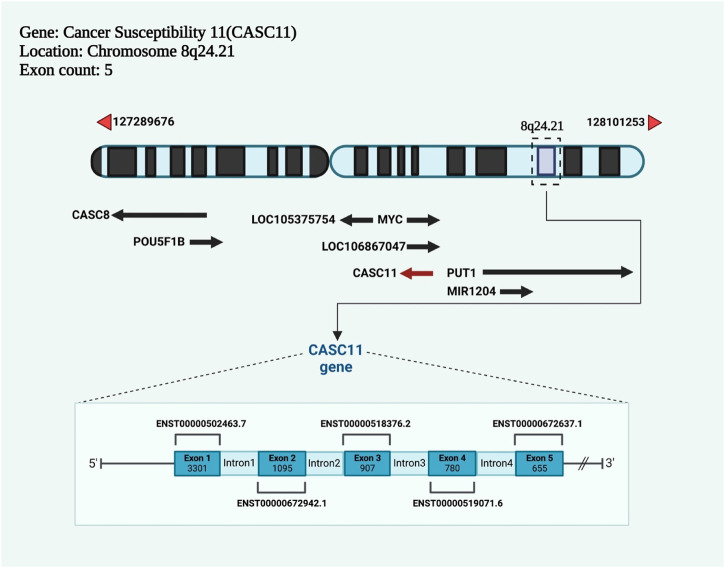
The newly discovered lncRNA cancer susceptibility 11 (CASC11) has a coding gene with five exons and is located on chromosome 8q24.21.

The coding gene of lncRNA cancer susceptibility 11 (CASC11) is an lncRNA encoded by a gene on chromosome 8q24.21 and has two transcript variants (https://www.ncbi.nlm.nih.gov/gene/100270680) (Figure 1). There are other *CASC* genes in the human genome such as *CASC1* (chr 12p12.1), *CASC2* (chr 10q26.11) and *CAS3* (17q21.1). Notably, CASC8 is also affiliated with the lncRNA class.

The expression of lncRNA CASC11 has been found to be elevated in different cancer types and the prognosis of the tumor is inversely correlated with the high CASC11 expression. As a result, lncRNA CASC11 has an oncogenic function in cancers. The biological characteristics of malignant cells, such as proliferation, migration, invasion, autophagy, and apoptosis can be controlled by this lncRNA. In addition to interacting with miRNAs, proteins, transcription factors, and other molecules, the lncRNA CASC11 modulates signaling pathways including Wnt/β-catenin and epithelial-mesenchymal transition (EMT) to carry out these regulatory functions (Zheng et al., 2021; Wang et al., 2022).

In this review, we have summarized studies on the role of lncRNA CASC11 in the carcinogenesis from cell lines, *in vivo*, and clinical perspectives. The data summarized in this manuscript highlights the importance of CASC11 in the carcinogenesis and suggests this lncRNA as a putative target for anti-cancer therapies.

### Role of CASC11 in cancers

#### Cell line studies

The role of CASC11 in the carcinogenesis has been evaluated in several cancer cell lines. In bladder cancer cell lines, upregulation of CASC11 has led to suppression of miR‐150 expression. However, miR‐150 overexpression could not affect expression of CASC11. Over-expression of CASC11 promotes, while miR‐150 overexpression inhibits cancer cell proliferation. In addition, miR‐150 could attenuate the increasing effect of CASC11 upregulation on proliferation of cancer cells. Conversely, upregulation of CASC11 could not affect migration and invasion of bladder cancer cells. Cumulatively, CASC11 has a role in regulation of proliferation of bladder cancer cells through modulation of miR‐150 levels (Wang et al., 2019).

Similarly, CASC11 has an oncogenic role in cervical cancer. In these cells, CASC11 silencing has inhibited proliferation, migratory potential and invasiveness and induced their apoptosis. Upregulation of CASC11 could facilitate cancer cell proliferation, migration and invasive abilities and suppress their apoptosis. Mechanistically, CASC11 promotes migration and invasion of cervical cancer cells through inducing activity of Wnt/β-catenin signaling (Hsu et al., 2019).

**FIGURE 2:**
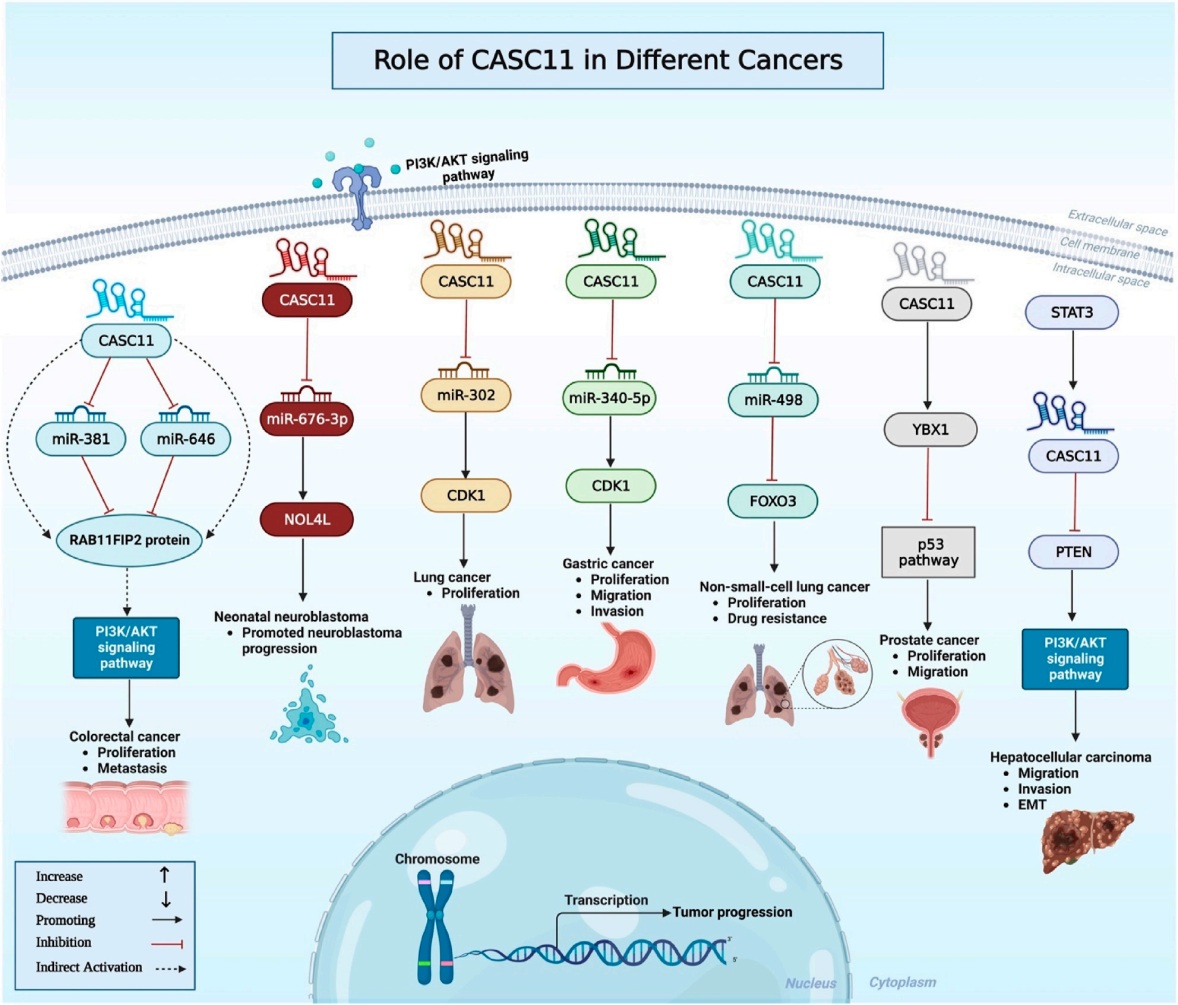
This diagram depicts the association between CASC11 and oncogenic signaling pathways in a variety of malignancies. CASC11 promotes tumor cell proliferation, invasion, migration, and survival by targeting specific genes like PTEN and YBX1 and sponging certain miRNAs. Some examples of these miRNAs are miR-381, miR-646, miR-676-3p, miR-340-5p, and miR-498.

Similar to bladder cancer, CASC11 has been shown to sponge certain miRNAs in colorectal cancer cell. Experiments in colorectal cancer cells have shown the ability of CASC11 to bind with miR-646 and miR-381-3p in the cytoplasm. Besides, miR-646 and miR-381-3p inhibitors could reverse the inhibitory effects of CASC11 knock out on proliferation of colorectal cancer cells. Notably, RAB11FIP2 has been found to be a common target of miR-646 and miR-381-3p. Mechanistically, CASC11 regulates PI3K/AKT pathway through regulation of miR-646 and miR-381-3p/RAB11FIP2 axis (Zhang et al., 2021). CASC11 can also enhance proliferation of colorectal cancer cells through targeting hnRNP-K and activating WNT/β-catenin signaling (Figure 2). Moreover, c-Myc has been shown to directly bind to the promoter of CASC11 and increase histone acetylation to induce expression of CASC11 (Zhang et al., 2016). CASC11 knockdown in esophageal cancer cells has led to enhancement of cell apoptosis. Moreover, its silencing has resulted in upregulation of expression of KLF6 protein. Based on the results of recovery experiments, CASC11 and KLF6 have been shown to be mutually regulated (Chen SG. et al., 2019). Another study in gastric cancer cells has shown that expression of CASC11 is induced by overexpression of LINC01116. Similarly, CASC11 overexpression has resulted in up-regulation of LINC01116. Both lncRNAs have important roles in induction of invasion and migration of gastric cancer cells (Su et al., 2019). CASC11 can also promote malignant features in gastric cancer through regulation of cell cycle pathway (Zhang et al., 2018).

In the glioma cells, CASC11 has been demonstrated to sponge miR-498 and increase expression of FOXK1 (Jin et al., 2019). Table 1 shows the results of cell line assays to determine function of CASC11 in various cancer types.

**TABLE 1:** Cell line assays to determine function of CASC11 in various cancer types (TCLs: tumor cell lines, NCL: normal cell line, ∆: knockdown or deletion, EMT: epithelial-mesenchymal transition).

Cancer type	Cell lines	Expression of CASC11 (TCLs vs. NCLs)	Interacting targets and regulators	Related signaling pathway	Function	References
Bladder cancer	TCLs: HT-1197, HT-1376	_	miR-150	_	↑CASC11	Wang et al. (2019)
					↑cell proliferation	
Cervical cancer	TCLs: HeLa, CaSki, SiHa, C-33A, MS751	Up	_	Wnt/β-catenin signaling pathway	∆CASC11 (in HeLa)	Hsu et al. (2019)
					↓cell proliferation, ↓migration, ↓invasion, ↑apoptosis	
	NCL: HEKn				↑CASC11(in CaSki)	
					↑cell proliferation, ↑migration, ↑invasion, ↓apoptosis	
Colorectal cancer	TCLs: SW480, SW620, LOVO, HCT116, RKO, Caco2, LS174T	_	miR-646 and miR-381-3p/RAB11FIP2	PI3K/AKT signaling pathway	∆CASC11	Zhang et al. (2021)
	NCL: FHC				↓cell growth, ↑G1 phase cell cycle arrest, ↓migration	
	TCLs: LOVO, SW480, SW620, M5, LS174T, RKO, HT29, HCT116, HEK293NCL: FHC	Up	c-Myc (regulator), hn-RNP-K	Wnt/β-catenin signaling pathway	∆CASC11	Zhang et al. (2016)
					↓cell growth and colony formation, ↑G1 phase cell cycle arrest, ↓migration	
					↑CASC11	
					↑proliferation, ↑migration	
Esophageal carcinoma	TCLs: OE19, OE33, TE-1, KYSE30, EC-109	Up	KLF6	_	∆CASC11	Chen et al. (2019b)
	NCL: HEEC				↓proliferation, ↑apoptosis	
Gastric cancer	TCLs: SNU-1, Hs746T	_	LINC01116	_	∆CASC11	Su et al. (2019)
					↓migration, ↓invasion	
					↑CASC11	
					↑migration, ↑invasion	
	TCLs: KATOIII, AZ521, MKN7	Up	miR-340-5p/CDK1	_	∆CASC11	Zhang et al. (2018)
	NCL: GES-1				↓proliferation, ↑apoptosis, ↑G0/G1 cell cycle arrest	
Glioma	TCLs: U87, U251, T98G, SHG44	Up	SP1 (transcriptional regulator), miR-498/FOXK1 axis	_	∆CASC11	Jin et al. (2019)
					↓proliferation, ↓migration	
Hepatocellular carcinoma	TCLs: Hep3B, Huh7, MHCC97h, SK-Hep-1, PLC/PRF/5, HCCLM3	Up	ALKBH5/UBE2T	_	∆CASC11(in Hep3B)	Chen et al. (2021)
					↓proliferation, ↓migration, ↓invasion	
	NCL: THLE-2				↑CASC11(in Huh7)	
					↑proliferation, ↑migration, ↑invasion	
	TCLs: SNU-398, SNU-182	_	miR-21	_	In carboplatin-treated TCLs	Liu et al. (2020)
					∆CASC11	
					↓cell viability (↑chemo-sensitivity)	
					↑CASC11	
					↑cell viability (↑chemo-resistance)	
	TCLs: SNU-398, SNU-182	_	miR-188-5p	_	↑CASC11	Cheng et al. (2019)
					↑proliferation	
	TCLs: HepG2, Hep3B, SMMC-7721, LM3	Up	STAT3 (transcriptional regulator), EZH2/PTEN	PI3K/AKT signaling pathway	∆CASC11	Han et al. (2019)
	NCL: L-02				↓migration, ↓invasion, ↓EMT (↑E-cadherin, ↓N-cadherin)	
	TCLs: HepG2, SMMC-7721	Up	YY1(regulator), EIF4A3/E2F1/PD-L1	NF-κB pathway, PI3K/AKT/mTOR signaling pathway	∆CASC11	Song et al. (2020)
	NCLs: THLE-3, HL-7702				↓cell viability, ↓colony formation, ↓PCNA (proliferation marker), ↓migration (↓MMP-2), ↓invasion, ↑apoptosis, ↓energy metabolism	
Lung cancer	TCLs: A549, H157, SPC-A-1 NCL: 16HBE	UP	miR-302/CDK1	_	∆CASC11	Tong et al. (2019)
					↓proliferation	
Neonatal neuroblastoma	TCLs: SK-N-AS, NB-1	Up	miR-676-3p/NOL4L	_	∆CASC11	Yu et al. (2020)
	NCL: hTERT-RPE1				↓cell viability, ↓migration, ↓invasion	
Non-small-cell lung cancer	TCLs: A549, H460, H1299, H322	Up	FOXO3 (regulator and target)/miR-498	_	∆CASC11	Yan et al. (2019)
	NCL: NHBE				↓proliferation, ↑G0/G1 cell cycle arrest, ↑apoptosis	
Ovarian cancer	TCLs: UWB1.289, UWB1.289+BRCA1	_	miR-182	_	↑CASC11	Cui et al. (2020)
					↑proliferation, ↓apoptosis	
Ovarian squamous cell carcinoma (OSCC)	TCL: UWB1.289	Up (chemotherapy drugs-treated TCLs vs. controls)	_	_	In chemotherapy drugs-treated TCLs	Shen et al. (2019)
					↑CASC11	
					↑cell viability (↑chemo-resistance)	
					∆CASC11	
					↓cell viability (↓chemo-resistance)	
Prostate cancer	TCLs: PC-3, DU145, 22Rv1, LNCaP	Up	YBX1/p53	p53 signaling pathway	∆CASC11	Sun et al. (2022)
					↓proliferation, ↓migration, ↓S phase cells, ↑G1 cell cycle arrest, ↓cyclinA2, CDK2, and CDK4 (G1/S phase-associated proteins)	
	NCL: RWPE-1				↑CASC11	
					↑proliferation, ↑migration, ↑S phase cells, ↑S cell cycle arrest, ↑ cyclinA2, CDK2, and CDK4 (G1/S phase-associated proteins)	
	TCLs: PC3, DU145, LNCaPNCL: PNT1a	Up	miR-145/IGF1R	PI3K/Akt/mTOR signaling pathway	↑CASC11	Capik et al. (2021)
					↑proliferation, ↑colony formation, ↑wound healing, ↑migration	
Small cell lung cancer	TCLs: SHP-77, DMS79, H345, DMS53, H446, H1341	_	TGF-β1	_	↑CASC11	Fu et al. (2019)
					↑stemness (↑CDD133+ cells)	

Obtained from https://app.biorender.com/biorender-templates. BioRender was used in accordance with the terms of the Academic License.

#### Animal studies

Consistent with *in vitro* studies, animal studies have affirmed the oncogenic role of CASC11. In animal models of cervical cancer, CASC11 silencing has led to reduction of tumor volume and weight and downregulation of β-catenin (Hsu et al., 2019). Similarly, experiments in animal models of colorectal cancer have shown the role of CASC11 in enhancement of tumor growth. Moreover, miR-646 and miR-381-3p inhibitors have been shown to reverse the inhibitory effects of CASC11 silencing on tumor growth and metastasis (Zhang et al., 2021). Besides, CASC11 silencing has reduced Ki-67 expression and suppressed metastases of colorectal cancer to lung and liver (Zhang et al., 2016). Other studies in animal models of glioma, hepatocellular carcinoma, lung cancer and prostate cancer support oncogenic role of CASC11 (Table 2).

**TABLE 2:** Animal models of cancer showing impact of CASC11 (∆: knockdown or deletion).

Cancer type	Animal model	Result	References
Cervical cancer	Male athymic nude mice	∆CASC11	Hsu et al. (2019)
		↓tumor volume, ↓tumor weight, ↓β-catenin	
Colorectal cancer	Female BALB/c nude mice	∆CASC11	Zhang et al. (2021)
		↓tumor growth, ↓tumor volume, ↓tumor weight, ↓Ki-67, ↓hepatic metastatic nodules	
	Male athymic BALB/c nude mice	∆CASC11	Zhang et al. (2016)
		↓tumor size, ↓Ki-67 (proliferation index), ↓lung metastasis, ↓hepatic metastasis	
Glioma	BALB/c nude mice	∆CASC11	Jin et al. (2019)
		↓tumor volume, ↓tumor weight, ↓migration cells	
Hepatocellular carcinoma	Male athymic BALB/c nude mice	↑CASC11	Chen et al. (2021)
		↑tumor volume, ↑tumor weight, ↑lung metastasis	
	Athymic nude mice	∆CASC11	Song et al. (2020)
		↓tumor growth, ↓lung metastasis	
Non-small-cell lung cancer	BALB/c nude mice	∆CASC11	Yan et al. (2019)
		↓tumor growth	
Prostate cancer	Male BALB/c nude mice	∆CASC11	Sun et al. (2022)
		↓tumor volume, ↓tumor weight, ↓tumor proliferation (↓Ki-67)	

#### Studies in clinical samples

Plasma levels of CASC11 has been found to be up-regulated, while levels of miR‐150 has been down-regulated in early stages bladder cancer compared with their levels in healthy controls. Notably, altered expressions of these two transcripts could separate patients with bladder cancer from healthy subjects. Moreover, CASC11 expression has been inversely correlated with miR‐150 expression in patients with bladder cancer but not in cancer-free subjects (Wang et al., 2019). In patients with cervical cancer, CASC11 expression has been positively associated with tumor size and FIGO staging and negatively correlated to the survival of patients (Hsu et al., 2019). CASC11 has also been found to be up-regulated in colorectal cancer tissues in association with tumor dimension, serosal invasion, metastasis to lymph node, and TNM stage (Zhang et al., 2016). Besides, expression of CASC11 in the esophageal carcinoma tissues has been remarkably higher than its expression in adjacent normal tissues. Up-regulation of CASC11 has been associated with higher pathological stage and lower overall survival rate in this cancer (Chen SG. et al., 2019). In gastric cancer tissues, expression of CASC11 has been found to be increased parallel with up-regulation of another lncRNA, namely, LINC01116. Expression levels of both lncRNAs have been higher in tissue samples with higher clinical stages (Su et al., 2019). Other studies that reported up-regulation of CASC11 in tumor tissues are shown in Table 3.

**TABLE 3:** CASC11 expression in clinical samples of cancer (PTTA: pairs of tumor tissues and adjacent normal tissues, TNM: tumor-node-metastasis, T stage: tumor stage, OS: overall survival, DFS: disease-free survival, FIGO: international federation of gynecology and obstetrics, TCGA: the cancer genome atlas, GEO: gene expression omnibus).

Cancer type	Samples	Expression of CASC11 (tumor vs. normal)	Kaplan-Meier analysis (impact of CASC11 up-regulation)	Association of high CASC11 expression with clinicopathologic parameters	References
Bladder cancer	Plasma samples from 89 patients and 62 controls	Up	_	_	Wang et al. (2019)
Cervical cancer	50 PTTA	Up	Poorer survival	Tumor size, FIGO stage	Hsu et al. (2019)
Colorectal cancer	27 PTTA	Up	_	Tumor size, lymph-vascular invasion, lymph metastasis, T stage	Zhang et al. (2021)
	36 PTTA	Up (in 32 out of 36 pairs)	_	Tumor size, serosal invasion, lymph metastasis, TNM stage	Zhang et al. (2016)
Esophageal carcinoma	45 PTTA	Up	Poorer survival	Pathological stage	Chen et al. (2019b)
Gastric cancer	76 PTTA	Up	_	Clinical stage, lymph node metastasis, distant metastasis	Su et al. (2019)
	80 PTTA	Up	_	Tumor size, lymph node metastasis, TNM stage	Zhang et al. (2018)
Glioma	35 PTTA	Up	Poorer OS	Tumor size	Jin et al. (2019)
Hepatocellular carcinoma (HCC)	72 PTTA	Up	Poorer OS	Tumor grade, metastasis	Chen et al. (2021)
	69 PTTA + patient blood samples	Up (tumor vs. normal and in blood samples: after carboplatin treatment vs. before treatment)	_	_	Liu et al. (2020)
	68 PTTA	Up	Poorer OS	_	Cheng et al. (2019)
	76 PTTA	Up (tumor vs. normal and tumor tissues with metastasis vs. without metastasis)	Poorer OS	_	Han et al. (2019)
	78 PTTA + serum of 78 patients and 40 controls	Up	Poorer OS and DFS	Maximal tumor size	Song et al. (2020)
Lung cancer	30 PTTA	Up	_	_	Tong et al. (2019)
Neonatal neuroblastoma	42 PTTA	Up	Poorer survival	_	Yu et al. (2020)
Non-small-cell lung cancer	40 PTTA	Up	Poorer survival	TNM stage, differentiation	Yan et al. (2019)
Ovarian cancer	64 PTTA + plasma samples from 64 patients and 58 controls	Up	Poorer OS	_	Cui et al. (2020)
Ovarian squamous cell carcinoma (OSCC)	Plasma samples from 72 patients and 56 controls	Up (patients vs. controls and in patients, after chemotherapy vs. before)	_	_	Shen et al. (2019)
Prostate cancer (PCa)	66 PTTA + TCGA and GEO datasets	Up	_	_	Sun et al. (2022)
	29 tumor tissues and 5 normal samples	Up	_	_	Capik et al. (2021)
Small cell lung cancer (SCLC)	Plasma samples from 71 patients and 54 controls	Up	Poorer OS	_	Fu et al. (2019)

## Discussion

CASC11 is an lncRNA participating in the pathoetiology of diverse cancers as well as atherosclerosis, coronary artery disease and postmenopausal osteoporosis. It is universally up-regulated in malignant tissues and cancer cell lines compared with controls. Therefore, CASC11 can be regarded as an oncogenic lncRNA. This observation has also been affirmed in xenograft models of different cancers. Mechanistical studies have shown the sponging effect of CASC11 on miR-150, miR-646, miR-381-3p, miR-340-5p, miR-498, miR-21, miR-188-5p, miR-302, miR-676-3p, miR-498, miR-182, and miR-145. Moreover, expression of CASC11 has been shown to be regulated by c-Myc, STAT3, YY1, and FOXO3. Therefore, a complex network exists between cancer-related transcription factors, CASC11 and miRNAs. Identification of further molecules being involved in this network would facilitate design of novel therapeutic options for cancer.

Since this lncRNA can be tracked in plasma, it is a possible novel biomarker for detection of cancer recurrence after accomplishment of appropriate therapies.

Moreover, up-regulation of CASC11 in tumor tissues has been related with poor prognosis and adverse clinicopathological characteristics such as metastasis, lymph node involvement, higher grades and advanced stages. Thus, CASC11 is a putative prognostic marker for diverse cancers.

Taken together, CASC11 is an oncogenic lncRNA with possible application as diagnostic and prognostic marker in cancer. Yet, three are several unsolved questions about the underlying mechanism of CASC11 up-regulation in cancers, possible impact of genetic polymorphisms on its function and activity, the role of epigenetic factors in its regulation and the interactions between CASC11 and other regulatory biomolecules. Finding the answers to these questions might facilitate design of novel therapeutic modalities for cancers.
